# A Sustainable Road Transport Decarbonisation: The Scenario Analysis of New Energy Vehicle in China

**DOI:** 10.3390/ijerph20043406

**Published:** 2023-02-15

**Authors:** Anqi Chen, Shibing You, Huan Liu, Jiaxuan Zhu, Xu Peng

**Affiliations:** 1 Economics and Management School, Wuhan University, Wuhan 430072, China; 2 Merchants Union Consumer Finance Company Limited, Wuhan 430070, China

**Keywords:** new energy vehicles, generalised Bass model, carbon reduction, carbon neutrality, scenario analysis

## Abstract

Due to the prosperous development of the economy, the emissions of carbon dioxide (CO_2_) and other greenhouse gases (GHGs) have intensified and attracted attention worldwide. China has set the “dual-carbon” aim to pursue sustainable development in the transport sector. Thus, this study created a generalised Bass model to forecast new energy vehicle (NEV) ownership by introducing a new factor, charging piles, to reflect the infrastructure effects. Using the improved model with the hypothesis of annual mileage, an empirical analysis was conducted with the subject of NEVs in China by using the NEV-related panel data from 2010 to 2020, and the forecast result is outstanding with a goodness-of-fit of 99.7%. With the forecasts, carbon emission reduction was calculated with a bottom-up method. To further discuss the pathway to achieve carbon neutrality in the transport sector of China, a scenario analysis was conducted with ideal, enhanced, and radical constraints. The results show that if all factors remain “as is” until 2050, China will be far from carbon neutrality. Thus, this paper proposes relevant policy implications to assist the government to obtain effective methods to assess carbon reduction benefits and find viable pathways to a sustainable road transport system.

## 1. Introduction

In March 2021, The Meteorological (Met) Office announced that CO_2_ levels in the atmosphere were 50% higher than when humanity began to burn fossil fuels at a large scale at the start of the industrial revolution [1]. The rising level of CO_2_ concentrations is the main attribute to global warming [2], which intensifies heatwaves, risks of drought, and extreme weather, all posing catastrophic threats to the global ecosystem [3]. The transportation sector accounts for 23% of the world CO_2_ emissions from burning fossil fuel, with over three quarters from road transport [4]. Thus, to eliminate CO_2_ emissions in the transport sector, the market of NEVs is gradually moving towards marketisation with the implementation of renewable energy resources and intelligence technologies. Motivated by the socio-economic background, the current context of carbon neutrality, and the gaps in the previous related studies, our study mainly focuses on the forecast of NEVs, energy transformation from traditional fossil fuels to renewable energies, and the carbon emission reduction potential in the industry, trying to propose effective methods for road decarbonization and generalise the methods to other countries with similar conditions.

As one of the top economies and the largest carbon emitter, China’s low-carbon transition is vital not only to its own development but to other countries around the world. Although China announced the “dual-carbon” aim to transition to green and low-carbon development [5], the total carbon dioxide emissions in China reached nearly 12 billion tons in 2021 with the transportation sector contributing 11% as one of the main culprits [6]. Facing such severe impacts, 193 parties, including China, have joined the Paris Agreement with the aim of limiting global warming to well below 2.0 °C, ideally below 1.5 °C [7]. The “dual-carbon” aim indicates achieving carbon peaking in 2030 and carbon neutrality in 2060. Therefore, to fulfil the aim of carbon neutrality by 2060, the transportation sector is a crucial sector of the economy requiring careful thought and consideration. The decarbonization of road transport will be an essential component in achieving carbon neutrality in the transportation sector, and a series of regulations and policies have been enacted in China to facilitate the shift from internal combustion engine vehicles (ICEVs) relying on fossil fuels, to new energy vehicles (NEVs). In this study, NEVs indicate vehicles powered by new energy sources other than fossil fuels and with new powertrains, including BEVs (Battery Electric Vehicles), PHEVs (Plug-in Hybrid Electric Vehicles), and FCVs (Fuel Cell Vehicles). BEVs indicate electric vehicles powered only by chemical energy stored in a rechargeable battery pack, without the use of other secondary energy sources. PHEVs indicate hybrid electric vehicles, which use a battery pack that can be recharged by plugging a charging cable into an external power source and can also be charged by a generator driven by the on-board internal combustion engine, and are partly nonpetroleum fueled vehicles. FCVs are vehicles that are driven by their own fuel cell motor. The fuel cell used in this type of vehicle is powered by electricity generated by the redox reaction between oxygen in the air and compressed hydrogen gas.

Although NEVs are considered to produce fewer carbon emissions due to the low usage of petrol, their effectiveness of decarbonization is still controversial as they generate CO_2_ indirectly by consuming large amounts of electricity, especially in China where electricity production is mainly based on thermal sources. In particular, to produce 1 unit (kWh) of electricity, 0.32 kg of coal or 0.312${\;m}^{3}$ of natural gas is combusted, generating 3.06 kg and 1.87 kg of CO_2_ emissions per unit combustion of coal and natural gas, respectively [8].

To validate the CO_2_ emission reduction capability of NEVs, previous studies have built a solid foundation for qualifying carbon reduction, but most of the research is limited to a certain type of NEVs such as BEVs or heavy-duty trucks [9], and the scope is usually worldwide or provincial scale with a top-down method. Thus, it is essential to analyse the CO_2_ emission reduction capability of NEVs in China with a bottom-up method. Using such a method, forecasting NEV ownership is the preliminary step. The previous studies for NEV ownership forecasts can be classified into two categories: (1) Forecasting methods using ICEVs as references, including logistic curve forecasting models and BP neural network models, etc., [10,11,12] and (2) factors influencing NEV ownership.

Previous research of NEV ownership influencing factors can be divided into three aspects. For consumer preferences, previous research mainly addressed factors including consumers’ values, financial status, concern for environmental issues, policy incentives, and relevant infrastructure [13,14,15,16,17,18]. However, from both the supply and demand side, the results indicate that potential consumers have the highest sensitivity to infrastructure, especially charging piles [19]. For policies and regulations, previous research mainly focused on price subsidies, tax incentives, and industrial policies. The early study of Gallagher and Muehlegger [20] proved that the type of tax incentives and generosity of the incentives play important roles in boosting NEV sales. Lin and Shi [13] demonstrated how policy measures might support the further growth of the NEV market, but the effectiveness varies at different stages of consumers’ intention. On the contrary, with the development of NEVs, specific research for the market in China shows that alternative policy tools can be used to replace the subsidy so that subsidy reduction in China is reasonable and feasible since subsidy stimulation was proved to be endogenous [21]. The research of Dong and Liu demonstrated that while the policy for the new energy vehicle industry (NEVI) in China continues to encourage government-guided consumption, industrial policies differ greatly. The effectiveness of subsidies will be reduced when the market-oriented NEVI gradually emerges [22]. For NEV-related technologies, a consistent conclusion in previous research is that battery technologies and charging technologies matter most. Specifically, Yi et al. studied the NEV market in China and found a positive correlation between the proportion of charging piles and NEV ownership [23]. By integrating the infrastructure of charging piles, it is proved that the diffusion of electric vehicles is closely correlated to the charging behaviours and the quantity of charging facilities [24].

As for the CO_2_ emission reduction capability of NEVs, numerous previous studies have explored the potential of GHG emission reduction ability of NEVs from the standpoint of Life cycle analysis (LCA) and the use phase. Although a few scholars still suspect the effectiveness of NEVs in mitigating GHGs, blaming the low market share of NEVs, the increasing emission of ICEVs, and the high dependence of battery technologies on thermal power generation for production and use [25,26], most previous studies proved that NEVs showed a positive performance in reducing CO_2_. Moriarty and Wang proved that an important means to reduce GHGs for road transport is to promote NEVs, particularly in urban areas [27]. Helmers also suggested that small-size BEVs are useful in mitigating CO_2_ emissions with the data of 59 countries and regions [28]. Palencia et al. constructed an approach with two steps to estimate the optimal market penetration of NEVs by setting scenarios based on availability of electric vehicles, the number of lightweight vehicles, and cost [29]. Specifically in China, a country with primary power generation of burning coal, the environmental benefits of adopting NEVs are controversial. In our previous research, we also compared emission reductions from NEV adoptions in 31 provinces in China and claimed that future emission reductions will mainly be contributed by the southern regions for their market potential and clean power [30]. Yang et al. assessed the CO_2_ emission in major cities in China under different scenarios and proposed a relevant road planning system [31,32].

As discussed above, for NEV ownership, previous studies emphasise the forecast method by implementing a basic Bass model, leading to a wide gap between the forecasted value and the true value. This study improves the forecast accuracy by introducing the impacts of infrastructure, especially charging piles in China, reflecting the real-world situation effectively. Another shortcoming of previous studies is that they fail to reflect the relevance between NEV ownership and carbon reduction, yet our study set up a bottom-up method to assess the carbon reduction potential of NEVs under the context of carbon neutrality. Further, previous studies are limited to pure electric vehicles or new energy passenger cars while our study expands the study subjects to provide a more comprehensive analysis of carbon reduction of NEVs including passenger cars, buses, and trucks.

To address the research objectives, whether NEVs can reduce carbon emissions or not and how to quantify the benefits, an improved forecasting method for NEV ownership was introduced based on the generalised Bass model to improve the accuracy of forecasts. Second, to verify the effectiveness of the model using a bottom-up approach, an empirical analysis was carried out by collecting China’s NEV market-related data and adjusting the slump in 2020 due to the unexpected outbreak of COVID-19. The improved forecasting method for NEVs and bottom-up approach to assess carbon reduction have strong generalisation ability, which can be implemented under the context in other countries. The study subjects include different types of NEVs, including BEVs (Battery Electric Vehicles), PHEVs (Plug-in Hybrid Electric Vehicles), and FCVs (Fuel Cell Vehicles) with different usage types (i.e., passenger cars, buses, and trucks). Further, the study also hypotheses the annual mileage based on historical data, and in the scenario analysis, varied average power consumption per vehicle, thermal power proportion, FCV rate, and UF ratio are set according to different scenarios. Third, this study evaluated the potential for China’s NEVs to reduce CO_2_ emissions from 2025 to 2050 combined with the four scenarios and combined with the ones introduced by the International Energy Agency (IEA). Since CO_2_ is the main culprit for global warming, the analysis can shed some light on how to slow down the elevating temperature. Finally, based on the above analysis, relevant policy recommendations are promoted to accelerate the process of carbon neutrality.

## 2. Materials and Methods

### 2.1. Data

As discussed above, to study the carbon reduction benefits of NEVs in the transport sector, China, a major carbon emitter with gigantic sample size and practical significance, is irreplaceable. Since the market penetrations vary among NEV technologies, this study mainly focuses on 3 NEV technologies, classified according to the China Association of Automobile Manufactures (CAAM), as BEVs, PHEVs, and FCVs, reflecting the existing mainstream electrification pathways for road vehicles in China.

The data of NEV stock was retrieved from the Traffic Administration Bureau of the Ministry of Public Security (TABMPS) in China [33]. The mileage data of NEVs was obtained from the “New Energy Vehicle Big Data Research Report” [34] and “New Energy Vehicle National Big Data Alliance Briefing” [35] released by the National Big Data Alliance of New Energy Vehicles (NDANEV), including the annual accumulative mileage classified by vehicle type (passenger cars, buses, and trucks) and NEV technologies (BEVs, PHEVs, and FCVs). We used the quantity of charging piles to indicate the level of NEV-related infrastructure construction, building on the strong foundation of previous studies. The China Electric Vehicle Charging Infrastructure Promotion Alliance (EVCIPA) provided the data of charging pile stock. All the data in the research was retrieved at an annual level from 2010 to 2020. To process the data, the unexpected strike of COVID-19 in 2020 was adjusted to better reflect the true value.

### 2.2. Methods

#### 2.2.1. Bass Model

Since 1960, a series of diffusion theories have been established to model the actual shape of new product adoption. Among them, the Bass model is a widely used method to describe and predict the growth trend, especially durable products, with the application of the innovation diffusion theory [36].

Potential buyers of a new product are divided into two types in the assumption of the Bass model: innovators and imitators. Innovators denote first-time buyers who are impacted simply by “external communication” such as advertisement, policies, or mass media. Imitators are people who are influenced simply by word-of-mouth communication or so-called internal influence from previous buyers [37].

When forecasting the ownership of NEVs, three parameters are implemented in the Bass model, namely (1) the maximum market potential, (2) the innovation coefficient, and (3) the imitation coefficient: (1) The maximum market potential, which is represented by m in the model, denotes the number of adopters when the new product is completely diffused. (2) The innovation coefficient, which is represented by p in the model, denotes the degree to which innovators receive external influence. (3) The imitation coefficient, which is represented by q in the model, denotes the degree of internal influence.

The basic expression of the Bass model is as follows:

(1) $$\frac{f\left(t\right)}{1-F\left(t\right)}=p+qF\left(t\right)$$

where $f\left(t\right)$ denotes the probability density function of buyers at time t, meaning the probability of buying at time t; $F\left(t\right)$ represents the cumulative density function of buyers at time t; p is the innovation coefficient; and q is the imitation coefficient.

From the first-order differential equation of Equation (1), the cumulative number of buyers at time t is:

(2) $$N\left(t\right)=mF\left(t\right)=m\frac{1-e^{-\left(p+q\right)t}}{1+\frac{q}{p}e^{-\left(p+q\right)t}}$$

where m is the maximum market potential.

#### 2.2.2. Improved Generalised Bass Model

The traditional Bass model does not consider external variables affecting market diffusion. Further, it is unable to explain the effects of several fundamental economic factors, which compromises the model’s accuracy and adaptability. Thus, a generalised Bass model is presented to address the drawbacks of the basic model [38], as shown in Equations (3) and (4):

(3) $$\frac{f\left(t\right)}{1-F\left(t\right)}=\left[p+qF\left(t\right)\right]x\left(t\right)$$

(4) $$F\left(t\right)=\frac{1-e^{-\left(p+q\right)X\left(t\right)}}{1+\frac{q}{p}e^{-\left(p+q\right)X\left(t\right)}}$$

where p is the innovation coefficient indicating first-time NEV buyers who are impacted by external factors, and q is the imitation coefficient indicating potential NEV buyers who are influenced by previous buyers. $x\left(t\right)$ is the external factor function, and $X\left(t\right)$ is the integral function of $x\left(t\right)$.

As discussed in the Introduction section, infrastructure, policy incentives, and NEV-related technologies are the primary factors influencing the promotion and use of NEVs in China. Among the above factors, the government’s NEV subsidy policies played a crucial role in the early stages of NEV diffusion, but they also put tremendous financial strain on the government. The Chinese government’s gradual reduction of subsidies shows that the market for NEVs has steadily transitioned from governmental stimulation to endogenous development. Hence, the primary focus in the study is on the construction of infrastructure. Considering that the business model of battery swapping is not mature yet, to examine the effects of infrastructure development, this study selects the number of charging piles as a main parameter.

According to the data from EVCIPA, the ratio of the NEV ownership to charging piles has gradually dropped from 9:1 in 2014 to about 3:1 in 2021. Although the pressure of charging has been relieved to a certain extent, there is still a wide gap from the ideal condition. Based on our previous study [30], to improve the generalised Bass model to fit under such conditions, adding the factor of charging piles was proved to be effective to improve the generalised Bass model, the expression of $x\left(t\right)$ can be written as:

(5) $$x\left(t\right)=1+\beta \frac{C\left(t\right)-C\left(t-1\right)}{C\left(t\right)}$$

where $C\left(t\right)$ is the number of charging piles at time t, and *β* is a coefficient that measures the promotion effect of infrastructure construction on the promotion and application of NEVs.

Integrating the above equation from 0 to t, we can obtain a continuous form of Equation (5):

(6) $$X\left(t\right)=t+\beta \frac{lnC\left(t\right)}{lnC\left(0\right)}$$

### 2.3. CO_2_ Emission Reduction Estimation (WTW Phase)

Life cycle assessment (LCA) is a methodology established to assess environmental impacts at all stages in the life cycle of a product from a qualitative perspective [38]. The WTW method, a simplified LCA, is the predominant technique to evaluate energy savings and GHG emissions in the transportation sector when evaluating policy alternatives or regulatory effectiveness [39,40,41]. The WTW approach is comprised of two stages: the well-to-tank (WTT) stage and the tank-to-well (TTW) stage. The WTT stage covers steps involved in producing fuel, including feedstock (petroleum, coal, natural gas, renewable energy) production, the transportation of feedstock to the fuel production site, and the operations between the supply of feedstock and the fuel production site. The TTW stage, which refers to the operation of the vehicle during the course of its lifetime, is concerned with gasoline distribution, which includes all actions between the fuel production site and the vehicle tank. In this study, distinct from the previous top-down method, carbon emissions are calculated based on a bottom-up method from the 2006 Intergovernmental Panel on Climate Change (IPCC) guide to comply with international conventions and to provide a thorough analysis. The total carbon emissions determined by the approach are equal to the sum of carbon emissions for each energy source by combining energy unit conversion, energy carbon emission factors, and converted energy consumption.

#### 2.3.1. The WTT Stage CO_2_ Emissions

BEVs are pure electric cars that only use rechargeable battery packs as their primary source of energy. The overall power consumption of BEVs is calculated as the sum of the electricity used by each vehicle and the transmission losses, which can be estimated by Equation (7):

(7) $$PC_{\mathrm{BEV}}=\frac{\beta_{\mathrm{BEV}}\times L}{\gamma_{tran}}$$

where $PC_{BEV}$ is the total power consumption of BEVs; $\beta_{BEV}$ is the power consumption per km; L is the driving distance; and $\gamma_{tran}$ is the power transmission efficiency.

Since PHEVs consume both gasoline and electricity, the calculation of the PHEV power consumption is different form BEVs. The utility factor (UF) of PHEVs is defined as the ratio of the charge-depleting (CD) range to the total driving distance, as shown in Equation (8). In general, the vehicle operates primarily on fuel in the charging sustaining (CS) range (i.e., the internal combustion engine), while on electricity (i.e., the battery) in CD range.

(8) $$UF=\frac{L_{E}}{L_{E}+L_{F}}=\frac{L_{E}}{L}$$

where UF is the utility factor of PHEVs; $L_{E}$ is the CD range; $L_{F}$ is the CS range; and L is the total driving distance.

Then, the power consumption of PHEVs can be estimated using Equation (9):

(9) $$PC_{\mathrm{PHEV}}=\frac{\beta_{\mathrm{PHEV}}\times L\times UF}{\gamma_{tran}}$$

where $PC_{PHEV}$ is the total power consumption of PHEVs, and $\beta_{PHEV}$ is the power consumption per km.

FCVs use electrochemical reaction to power their onboard electric motors by generating electricity using oxygen from the air and compressed hydrogen without carbon emission. Thus, the power consumption of FCVs is 0, that is, $PC_{PHEV}=0$.

The total power consumption of NEVs is the sum of the total power consumption of BEVs, PHEVs, and FCVs, which can be estimated using Equation (10):

(10) $$PC_{\mathrm{NEV}}=PC_{\mathrm{BEV}}+PC_{\mathrm{PHEV}}+PC_{\mathrm{FCV}}=PC_{\mathrm{BEV}}+PC_{\mathrm{PHEV}}$$

China mainly relies on thermal power generation to produce electricity. Equation (11) can be used to estimate the CO_2_ emissions related to NEVs in electricity generation, that is, WTT CO_2_ emissions:

(11) $$E_{\mathrm{WTT},\;\mathrm{NEV}}=PC_{\mathrm{NEV}}\times \alpha_{TP}\times \sum_{1}^{2}A_{i}H_{i}\left(c_{1i}+c_{2i}\right)$$

where $E_{WTT,\;NEV}$ is the CO_2_ emission in the WTT stage; $\alpha_{TP}$ is the share of thermal power generation in China; $A_{i}$ is the share of coal and natural gas in thermal power generation; $H_{i}$ is the amount of fuel required to produce 1 k Wh electricity; $c_{1i}$ is the CO_2_ emissions during fuel production; $c_{2i}$ is the CO_2_ emissions during fuel combustion; and i=1, 2 represents coal and natural gas, respectively.

Equation (12) estimates the emissions of CO_2_ from the production of fuel consumed by ICEVs:

(12) $$E_{\mathrm{WTT},\mathrm{ICEV}}=\beta_{\mathrm{ICEV}}\times L\times c_{1}$$

where $\beta_{ICEV}$ is the fuel consumption per km of ICEVs; L is the driving distance; and $c_{1}$ is the CO_2_ emissions in the fuel production.

#### 2.3.2. The TTW Stage CO_2_ Emissions

Since BEVs and FCVs do not release CO_2_ in the driving process, there is no CO_2_ emission during the TTW stage. For PHEVs, at the CS range, it can be regarded as the ICEVs, resulting in CD range mattering the most. Therefore, the CO_2_ emission of NEVs in the TTW stage is zero, that is, $E_{TTW,NEV}=0$.

The CO_2_ emission of ICEVs comes from the combustion of fuel, and it can be estimated using Equation (13):

(13) $$E_{\mathrm{WTT},\mathrm{ICEV}}=\beta_{\mathrm{ICEV}}\times L\times c_{2}$$

where $c_{2}$ is the CO_2_ emissions from fuel combustion.

After estimating the WTT and TTW CO_2_ emissions of NEVs and ICEVs, we can obtain carbon emission reduction of NEVs using Equation (14):

(14) $$CR_{\mathrm{WTW}}=E_{\mathrm{WTT},\mathrm{ICEV}}+E_{\mathrm{TTW},\mathrm{ICEV}}-E_{\mathrm{TTW},\mathrm{NEV}}$$

where $CR_{WTW}$ is the CO_2_ emission reduction of NEVs during the WTW phase.

## 3. Results and Discussion

### 3.1. NEVs Ownership from 2025 to 2050

With the rapid evolution of statistical theory and computer technologies, academics tend to use various intelligent algorithms to estimate coefficients of Bass models or generalised Bass models such as machine learning [42], sequential quadratic programming algorithms [43], and genetic algorithms [44], etc. The genetic algorithm, one of the frequently used algorithms, can be easily parallelised and has strong robustness and global search capabilities, so in this study, the genetic algorithm was chosen to estimate coefficients of the generalised Bass model.

The model estimated maximum market potential m, innovation coefficient p, imitation coefficient q, and coefficient β. The maximum market potential m can either be exogenously provided based on theoretical analysis or derived from the Bass model. An issue to be pointed out is that the innovation coefficient p is highly sensitive to m, so that the estimation of m significantly affects that of p. Thus, integrated with previous relevant research, this study sets m within the range between 500 and 700 million based on relevant literature [45,46,47] instead of estimating m through the Bass model. The coefficient estimation result is shown in Table 1.

**Figure 1:**
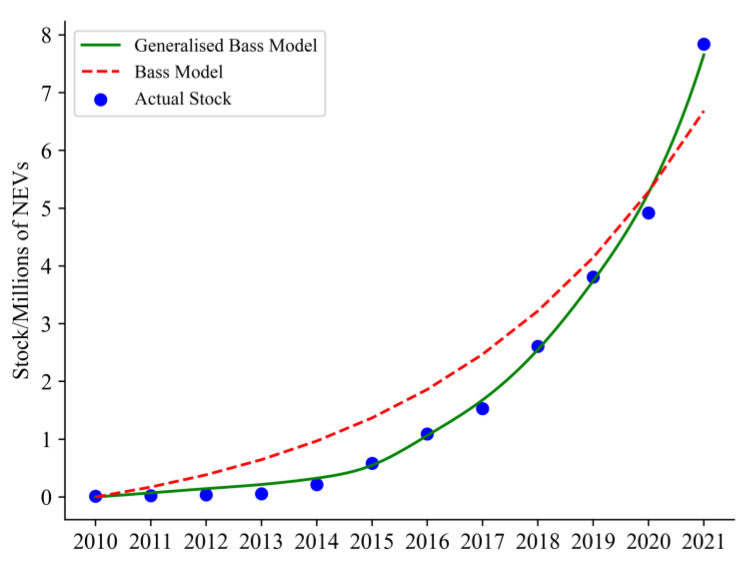
Comparison for Results of Improved Generalised Bass Model and Bass Model.

The results demonstrate that the imitation effect dominates the diffusion of NEVs, consistent with the general diffusion process, as the innovation coefficient p is substantially smaller than the imitation coefficient q. β being 2.50, indicating that in the process of NEV diffusion, the construction of infrastructure has promoted the diffusion and application of NEVs to a certain extent. $R^{2}$ is 0.997, which indicates that the improved generalised Bass model established in this study has a high accuracy in the projection of the ownership of NEVs. The improved generalised Bass model was outstanding as it depicted continuous improvement in infrastructure to represent the improvement of infrastructure development, as is shown in Figure 1.

This study compares the results of the improved generalised Bass model with the basic one in forecasting NEV ownership with the aim to show the validity of the improved model, as illustrated in Figure 1. With an R^2^ of 0.925, the improved generalised Bass model is more accurate than the basic one at predicting NEV ownership. The explanation for the high accuracy can be found in an accurate depiction of how infrastructure is always being improved to support the adoption and use of NEVs. As shown above, the traditional basic Bass model fails to take the impact of external factors or shocks into account, yet the new energy industry is still under initial development, and policy and market changes usually cause frequent impacts on the NEV industry, resulting in a poor fit of the basic Bass model to the dataset. In contrast, the poor fit condition is better addressed with the improved generalised Bass model. By introducing the degree of infrastructure development into the generalised Bass model, factors influencing NEV ownership are more accurately reflected. It solves the problem that the basic Bass model fails to explain the effects of some basic economic variables by integrating external variables during the diffusion of NEVs. Thus, the improved generalised Bass model improves the accuracy and flexibility of NEV ownership forecasting.

According to the coefficient estimation results in Table 1, the NEVs ownership (Million) in China at time t is:

(15) $$N\left(t\right)=593.71\times \frac{1-e^{-\left(1.53\times 10^{-5}+0.246\right)[t+2.5\frac{lnC\left(t\right)}{lnC\left(0\right)}]}}{1+\frac{0.25}{1.53\times 10^{-5}}e^{-\left(1.53\times 10^{-5}+0.25\right)[t+2.5\frac{lnC\left(t\right)}{lnC\left(0\right)}]}}$$

**Figure 2:**
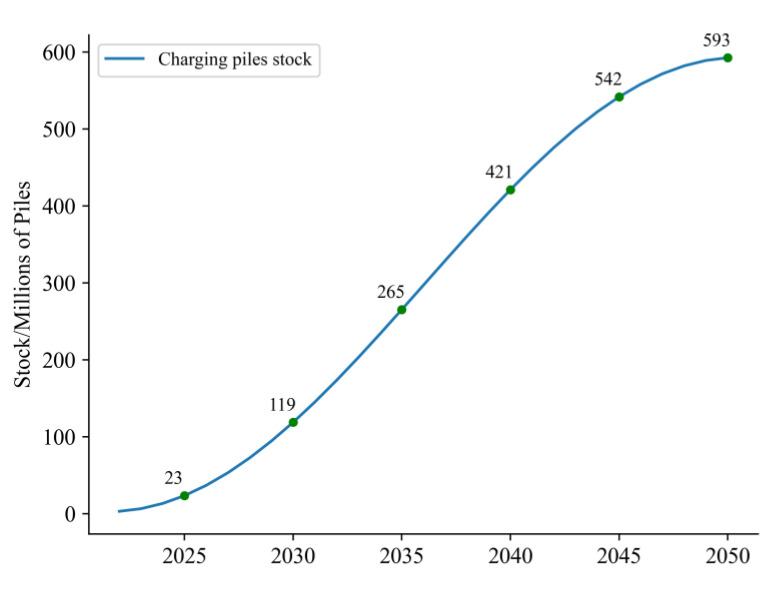
The Number of Charging Piles from 2020 to 2050.

It is important to estimate the number of charging piles before estimating the number of NEVs. According to the Chinese government’s New Energy Automobile Industry Development Plan, the goal of the vehicle–pile ratio is 1:1. The charging piles figure is depicted in Figure 2 combining the maximum market potential and the change curve of charging piles, which is represented as an S-shaped growth trend.

From Figure 2, although the quantity of charging piles continues to increase, the vehicle–pile ratio is still far from the ideal 1:1. The expansion of NEVs has outpaced infrastructure construction from the beginning of the diffusion. Ensuring the construction of charging stations, battery swap stations, and hydrogen refuelling stations is of utmost importance and can prevent negative effects on the diffusion process because the process will be hampered if related infrastructure is not built ahead of the NEV markets.

**Figure 3:**
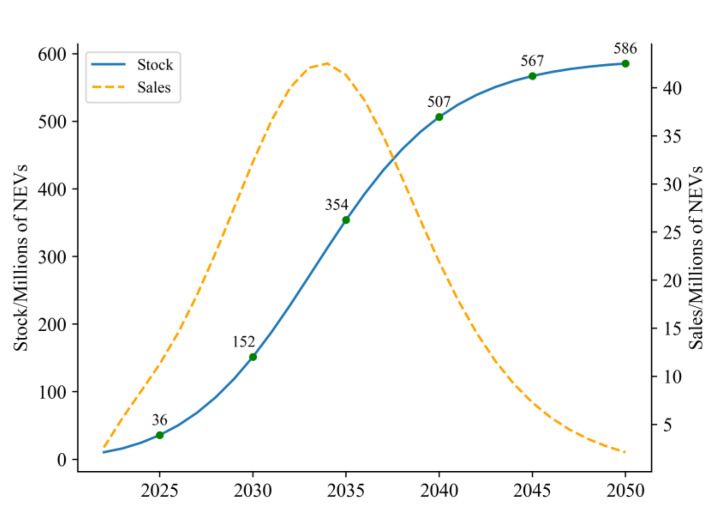
Forecast of NEV ownership from 2025 to 2050.

Hence, the quantity of NEV ownership can be effectively projected, and the result is shown in Figure 3.

Compared with previous studies, which mainly focuses on NEV sales and penetration, a typical study performed by Liu et al. introduced an optimized fractional discrete grey power model with the predicted results of China’s NEV sales to be 8.84 million in 2025, which is more conservative than our study [48]. Liu et al. used deep learning technology to forecast the NEVs indicating the NEV market will reach the 20% penetration goal in 2025, which is in accordance with our study while we provided a more specific figure instead of simply a percentage of market share [49]. Figure 3 shows that the marketization stage (now–2030), the rapid development stage (2031–2045), and the maturity stage (2045–2050) are three distinct stages that the NEV market in China will go through as it develops. Between now and 2030, NEVs in China will gradually transition from policy stimulation to endogenous development. Industrial technology will advance in this period in terms of critical performance metrics, yet battery energy density and NEV range will not have reached high-end levels. Additionally, the related infrastructure, such as charging piles, switching stations, and hydrogen refuelling stations, will not have been constructed to fully meet the demand of NEV market development. The years 2030–2045 will witness a rapid development of NEVs. At this stage, the NEVs’ relevant technology will have been developed and matured, and the supporting infrastructure will have also reached an ideal level. Under relevant promotion policies, ICEVs will be gradually withdrawn from the automobile market while NEVs will experience explosive growth. From 2045 onwards, the NEV market in China will enter a mature era. Along with the growth in population and city sizes, the market will gradually become saturated. By then, the number of NEVs in China will reach a level of above 550 million, entering a phase of development at a slow pace.

### 3.2. Scenario Analysis of CO_2_ Emission Reduction

A technique to forecast the expected value of performance indicators, occurrence of different situations, and related changes in the values of system parameters is a scenario analysis [50] under an uncertain environment given a time period. It has been proved to be effective for exploring possible or feasible ways of accomplishing environmental aims [51]. By conducting a scenario analysis, impacts of possible future events will be analysed in the study with the consideration of various alternative outcomes and to present different options for future carbon neutrality paths.

#### 3.2.1. Scenario Setting

With the constraint boundary of a 2030 carbon peak and 2060 carbon neutrality in China, combined with the IEA Scenarios [52], the corresponding energy structure target, technological advancement, and quantity of FCVs are calculated to form four different scenarios: Business As Usual (BAU), Ideal, Enhanced, and Radical as shown in Table 2.

(1) Business as Usual (BAU) Scenario

The baseline scenario, or BAU scenario, is used as a benchmark when comparing the corresponding effects of different technical and policy scenarios. It is set with the assumption that technology and policy will remain unchanged until 2050. Under such a scenario, only the parameter of NEV ownership will change with time, while other parameters will maintain in accordance with the data in 2020. According to the data on the accumulated mileage of NEVs, it is calculated that the average annual mileage of passenger cars, buses, and trucks in 2020 is 14.40 thousand kilometers, 34.52 thousand kilometers, and 14. 288 thousand kilometers, respectively. The driving distance per NEV is influenced by user demand, which has historically remained steady; it is anticipated to remain stable. Hence, average annual mileage increases with the ownership of NEVs.

(2) Ideal Scenario

The transport sector is in line with the stated policies, and new energy car development goals are set with the New Energy Automobile Industry Development Plan (2021–2035). In this scenario, constraints are relatively loose. The average power consumption of electric passenger cars, buses, and trucks will be down to 13.15 kWh/100 km, 63.28 kWh/100 km, and 29.06 kWh/100 km in 2030 and 9.72 kWh/100 km, 46.77 kWh/100 km, and 21.48 kWh/100 km in 2050, respectively, with the assumption that the average power consumption will decrease 1.5% per year [53]. According to the Stated Policies Scenario of IEA, the electricity structure will reach the indicative target of 40% from renewable sources by 2030 and 70% by 2050. Since FCVs purely rely on electricity generated from the electrochemical reaction of hydrogen fuel cells, they are “zero emission” new energy vehicles. The adoption, promotion, and application of FCVs can contribute to carbon reduction and carbon neutrality of road transport in China. Thus, the proportion of new FCVs in sales is expected to be 20% in 2030 and 50% in 2050. Another factor to be considered is the Utility Factor (UF) of PHEVs [54]. Compared with the US, EU countries, and Japan, the UF of PHEVs in China is at a low level because most users choose PHEVs simply for convenience of a new energy license plate, and the electric range restricts the UF [55]. In this scenario, the UF is set to be 0.69 in 2030 in line with the US level and 0.8 in 2050 in line with the EU countries’ level.

(3) Enhanced Scenario

According to the Sustainable Development Scenario of IEA, average power consumption ($\beta_{E}$) will be down to 12 kWh/100 km, buses down to 57.73 kWh/100 km, and trucks down to 26.51 kWh/100 km in 2030, and passenger cars down to 7.37 kWh/100 km, buses down to 35.51, and trucks down to 16.31 kWh/100 km in 2050. As for the electricity structure, 50% of electricity will be generated by clean energy in 2030, and 80% in 2050. Combined with the IEA scenario, FCVs will be 40% in truck sales in 2030 and 75% in 2050. In this scenario, the UF is set in line with EU countries’ level at 0.8 in 2030 and 1 in 2050.

(4) Radical Scenario

Because of technical restrictions, the average power consumption of different types of NEVs is assumed to remain in line with ideal scenarios. Integrated with the Net Zero Emissions Scenario of IEA, renewable energy will account for 60% of electricity output in 2030 and 90% in 2050, and FCVs will be 50% in truck sales in 2030 and 100% in 2050. In this scenario, PHEVs will all be replaced by BEVs by 2030, equivalent to the situation that UF = 1.

#### 3.2.2. CO_2_ Reduction Potential under Different Scenarios

The CO_2_ reduction can be calculated with the following equation:

(16) $$CR_{WTW}=\overset{3}{\underset{i=1}{\sum }}N_{i}L_{i,avg.}\beta_{F}\left(c_{1}+c_{2}\right)-\overset{3}{\underset{i=1}{\sum }}N_{i}L_{i,avg.}\beta_{E}\alpha H\left(c_{1}+c_{2}\right)/\gamma$$

where $N_{i}$ is NEVs ownership, $L_{i,avg.}$ is annual average mileage (for PHEVs, this figure should multiply with UF), $\beta_{F}$ is the average fuel consumption (L/100 km), $\beta_{E}$ is average power consumption (kWh/100 km), α is the proportion of thermal power electricity, *H* is the fuel required to produce per degree of electricity, $c_{1}$ and $c_{2}$ are CO_2_ emissions from fuel combustion and production processes, and γ is the efficiency of electrical energy transmission.

**Figure 4:**
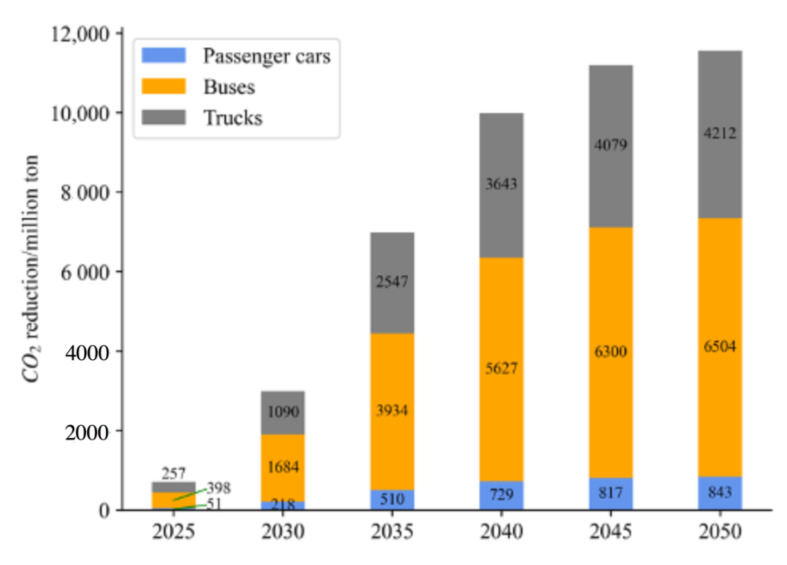
CO_2_ emission reduction potential under BAU.

Under BAU, according to the forecast of NEVs in the previous section, the CO_2_ reduction potential can be calculated as shown in Figure 4.

When all factors, especially policies and technologies, remain as is in 2020, under such scenario, NEVs will have reduced carbon emissions by 1159.51 million tonnes. It is considerably different from carbon neutrality when compared to the projected 2601 million tonnes of CO_2_ emissions in China. Therefore, it is critical that the transportation industry take additional steps to encourage the use of NEVs.

**Figure 5:**
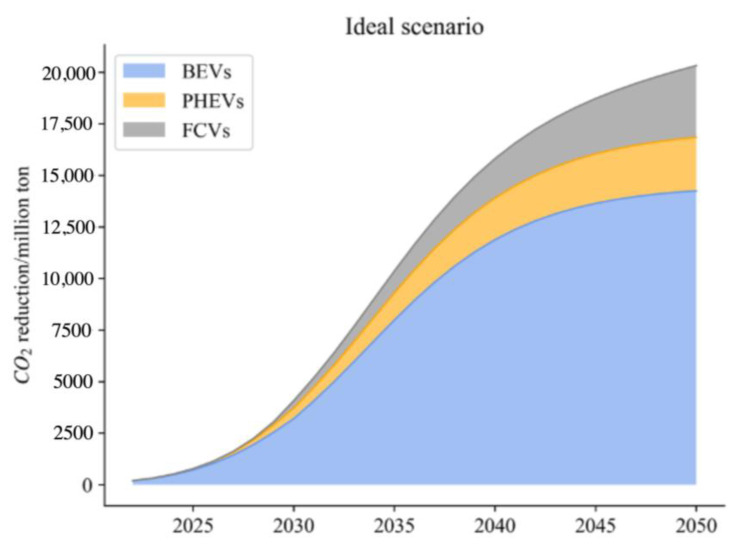
Carbon reduction under the ideal scenario.

**Figure 6:**
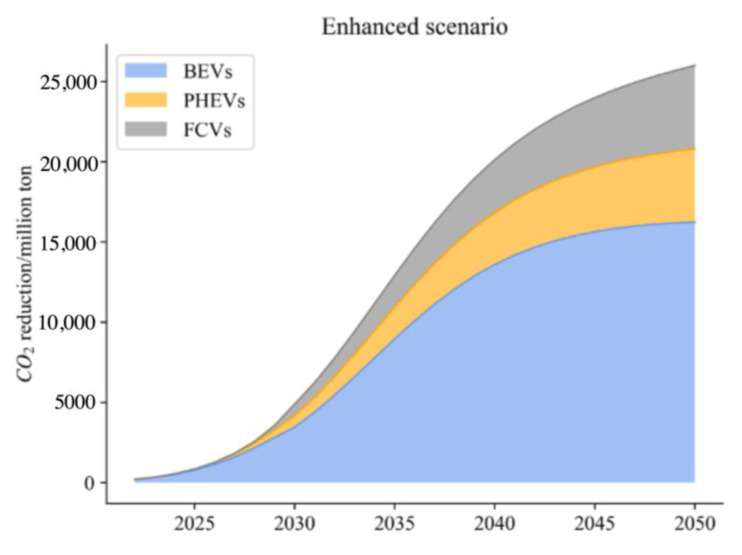
Carbon reduction under the enhanced scenario.

**Figure 7:**
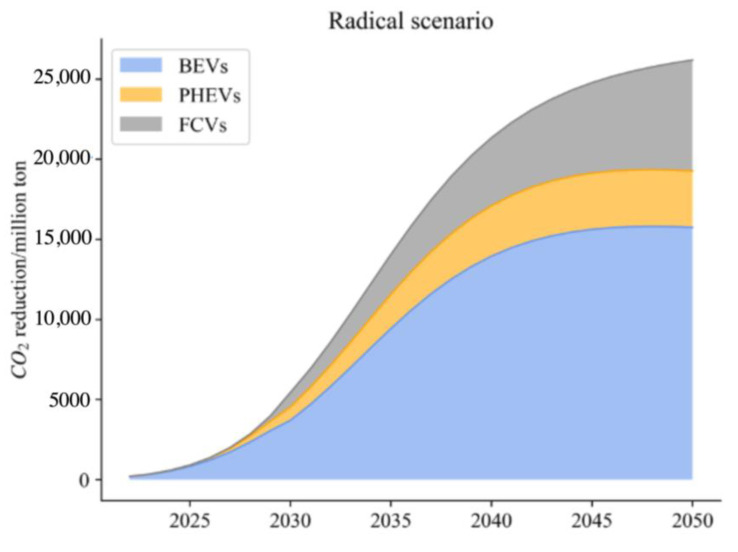
Carbon reduction under the radical scenario.

From the simulation, compared with previous research results as shown in Figure 5, Figure 6 and Figure 7, the results and scope of the predicted carbon reduction under different scenarios are within a reasonable range. Thus, our results on energy consumption and CO_2_ emissions are believed to be within a credible interval. To address the impact of uncertainties related to different parameters, we conducted a sensitivity analysis based on the baseline scenario to evaluate the variations in thermal power proportion and traveling distance.

From the above analysis, with the reduction of the thermal power ratio from the baseline scenario to 40%, the CO_2_ reduction potential of NEVs increases significantly. Thus, compared to the values in the BAU, a cleaner power structure can foster the process of carbon neutrality. As for the travelling distance (TD), more frequent use of NEVs contributes to reduce energy consumption compared with the traditional use of ICEVs, thereby reducing carbon emissions. Although the figure is not noticeable when TD increases to 40%, the carbon reduction potential is noticeable when TD increases above 60%. Comparing three distinct scenarios, it can be seen that under the ideal scenario, reducing the average electricity consumption of BEVs and PHEVs contributes most to CO_2_ emission reduction. It is followed by reducing the proportion of thermal power electricity, which ranks second. In contrast, increasing UF of PHEVs and promoting FCVs in heavy truck transport is not significant due to over 80% of vehicles being dominated by BEVs, and the turnover in road transport is higher for passenger cars and buses. Under enhanced and radical scenarios, carbon emission reduction does not improve significantly. It is worth noting that phasing out thermal power electricity will make an outstanding contribution to CO_2_ reduction in the transport sector. Reducing average electricity consumption of BEVs and PHEVs also has certain contributions, while surprisingly the contribution of full application of FCVs for trucks is very limited.

Table 3 provides the CO_2_ emission reduction potential by different usage types of vehicles. Although the quantity of NEV buses ownership and total mileage is relatively low, the corresponding ICEV buses consume more fuel and therefore contribute the most to reducing CO_2_ emissions. Further, passenger cars with the highest share of ownership and cumulative mileage contribute more than 37% of the CO_2_ emission reduction. Trucks also play an outstanding role in CO_2_ emission reduction as ICEV trucks have high-energy consumption.

## 4. Policy Implications

From our analysis above, NEVI would go through three distinct stages, from marketization to the maturity stage, starting with urban regions and eventually diffusing to rural areas. In 2050, the quantity of NEV ownership in China will exceed 5.86 million.

According to the calculations under different scenarios, under BAU, carbon reduction will be far from carbon neutrality, meaning that the government should implement pertinent policies and encourage technological development to accelerate the diffusion of NEVs. Under an ideal scenario, reducing the average electricity consumption of BEVs and PHEVs will contribute most to CO_2_ emission reduction, while from enhanced to radical scenarios, CO_2_ emission reduction does not improve significantly. It is worth noting that phasing out thermal power electricity makes an outstanding contribution to CO_2_ reduction in the transport sector. Reducing average electricity consumption of BEVs and PHEVs also has certain contributions, while, surprisingly, the contribution of full application of FCVs for trucks is very limited under all three scenarios. From the perspective of NEV types, BEVs account for the largest share in the Chinese NEV market and obtain the highest capability in reducing CO_2_ emissions. PHEVs account for nearly one fifth of the entire market, but their contribution is relatively small due to the low CS range. FCVs also make a small contribution to emission reduction due to the low ownership figure, but they have great potential for future emission reduction since they do not produce any CO_2_ emissions in the electricity production process. In terms of the market segmentation of new energy vehicles: although the ownership and cumulative mileage of new energy buses is small, they contribute the most to CO_2_ emission reduction due to the high CO_2_ emissions of fuel buses. Passenger cars and trucks will also have an outstanding effect on CO_2_ emission reduction.

This study also proposes some pertinent policy implications based on the results and can be implemented not only in China but in other countries worldwide. First, the government should introduce more policy support for the NEV industry during the marketization phase, especially to boost the diffusion of NEVs in urban areas as a starting point. As discussed, the innovation coefficient, representing the intensity of policy support, has a strong degree of influence in the marketization stage of NEVs. However, China’s present NEV-related policy support diminishes with time, which will impede the development of the NEV market to a certain extent. Thus, during the marketization stage, governments should continue to provide support, such as offering tax incentives, purchase subsidies, vehicle licensing policies, etc., to promote the rapid diffusion process. Second, key technologies of core components of NEVs should be upgraded with increasing investments in science and technological innovation. As technology is critical in the mid to late stages of the development of NEVs and can advance the NEVs’ peak year, facilitating the carbon peak aim is critical in the transport sector. To elaborate, under the conditions where the source of electricity is of high emissions, the electricity consumption of BEVs causes their CO_2_ emissions in the WTT phase, so by reducing electricity consumption, the carbon emissions of road transport can be reduced to a large extent. Further, during the penetration process of NEVs, especially the initial stage, the government should take more measures related to information policy instruments (i.e., education and environmental publicity) to raise people’s conscientiousness of environmental protection and encourage more buyers to choose NEVs other than traditional ICEVs [56]. Another effective method to encourage current ICEV owners to transfer to NEVs is to promote a replacement policy by providing buyers subsidies or discounts to boost the demand of NEVs. In addition, the CS range of PHEVs in China is relatively low, so increasing the UF can reduce the fuel consumption and enhance the carbon reduction potential of PHEVs. As for FCVs, although they are completely clean vehicles, promoting them only has a limited contribution, so at the current stage it is not urgent to facilitate the application of FCVs. The government is encouraged to test the efficiency of FCVs in major urban cities to maximize their potential for subsequent development. Last, the electricity structure should be improved to gradually phase out thermal power replaced by renewable energies. As discussed in the scenario analysis, if a society heavily relies on thermal power, such as China, even if road transport is fully electrified, the carbon emissions are still beyond imagination. Therefore, with an aim of achieving carbon neutrality, apart from promoting NEVs, the electricity structure should be reformed to be dominated by renewable energies, reducing CO_2_ from the root cause.

## 5. Conclusions

This study enhances the generalised Bass model to propose a useful forecasting technique. Because the proposed method incorporates the effects of infrastructure building through the parameter, i.e., the number of charging piles, to boost forecast effectiveness, the performance of the proposed model employed in the paper outperforms previous methods. With a goodness-of-fit of 99.7%, the prediction model achieves exceptional results for the NEV ownership forecast in China. Further, this paper also conducts a quantitative analysis of NEVs’ potential to reduce emissions. NEVs have a far greater ability to reduce carbon emissions at the WTW phase when compared to ICEVs.

There are mainly three contributions of the study: (1) This study improves the accuracy of NEV ownership forecast by introducing the impacts of infrastructure, which can better reflect the real-world situation. (2) Under the carbon neutrality context, this study evaluates the carbon reduction potential of NEVs in different scenarios. (3) This study expands the study subjects to provide a more comprehensive analysis of carbon reduction of NEV passenger cars, buses, and trucks, with strong adaptation and generalization ability. However, we acknowledge that the research presented in this article is just a starting point, and the NEV ownership forecast model still has room for improvement. First, the forecasts in this paper based on the generalised Bass model can be further discussed with quantitative comparisons to other models for their effectiveness. Further, ICEVs as a substitute for NEVs can also influence consumers’ purchase willingness, so the relative price and driving cost of NEVs can be introduced in the improved generalised Bass model, laying a more solid microfoundation for this study.

## Figures and Tables

**Table 1:** Improved Generalised Bass Model Coefficient Estimation Results.

p	q	β	$R^{2}$
$1.53\times 10^{-5}$	0.25	2.50	0.997

**Table 2:** Design of Scenarios.

	2030				2050			
	Decreasing Rate of $\beta_{E}$	Proportion of Thermal Power Electricity (α)	FCV Rate	UF	Decreasing Rate of $\beta_{E}$	Proportion of Thermal Power Electricity (α)	FCV Rate	UF
Ideal Scenario	14.03%	60%	20%	0.69	36.45%	30%	50%	0.8
Enhanced Scenario	21.57%	50%	40%	0.8	51.83%	20%	75%	1
Radical Scenario	21.57%	40%	50%	1	51.83%	10%	100%	1

**Table 3:** Carbon emission reduction potential by different usage types of vehicles.

Million Tons									
Scenarios	Ideal			Enhanced			Radical		
Types	Cars	Buses	Trucks	Cars	Buses	Trucks	Cars	Buses	Trucks
2025	59	475	251	63	528	261	68	575	270
2030	294	2476	1311	339	2980	1580	384	3462	1602
2035	742	6406	3222	891	8083	3946	992	9178	3901
2040	1123	9872	4822	1384	12,782	5968	1504	14,105	5738
2045	1322	11,771	5638	1641	15,335	7020	1743	16,477	6564
2050	1425	12,827	6073	1768	16,644	7594	1838	17,455	6905

## Data Availability

The datasets generated for this study are available from the Traffic Administration Bureau of the Ministry of Public Security (TABMPS) in China. The mileage data of NEVs was obtained from the “New Energy Vehicle Big Data Research Report” and “New Energy Vehicle National Big Data Alliance Briefing” released by the National Big Data Alliance of New Energy Vehicles (NDANEV). The data of charging pile stocks and the charging data were obtained from the China Electric Vehicle Charging Infrastructure Promotion Alliance (EVCIPA).

## Abbreviations

NEV	New Energy Vehicles
BEV	Battery Electric Vehicles
PHEV	Plug-in Hybrid Electric Vehicles
FCV	Fuel Cell Vehicles
ICEV	Internal Combustion Engine Vehicle
SCC	The Social Cost of Carbon
WTW	Well-to-Wheel
WTT	Well-to-Tank
TTW	Tank-to-Well
CD range	Charge-depleting range
CS range	Charge-sustaining range
UF	Utility Factor
